# Integrated genomic and transcriptomic analysis of polydactyly in chickens

**DOI:** 10.3389/fvets.2025.1592068

**Published:** 2025-05-21

**Authors:** Zhiying Huang, Xiaomeng Miao, Diyan Li, Jia Liu, Haolin Chen, Yuan Su

**Affiliations:** ^1^College of Animal Sciences, Shanxi Agricultural University, Taigu, China; ^2^Institute of Animal Husbandry and Veterinary Medicine, Guizhou Academy of Agricultural Sciences (CAAS), Guiyang, Guizhou, China; ^3^School of Pharmacy, Chengdu University, Chengdu, China; ^4^Guizhou Province Livestock and Poultry Genetic Resources Management Station, Guiyang, China; ^5^Shanxi Key Laboratory of Animal Genetics Resource Utilization and Breeding, Shanxi Agricultural University, Taigu, China

**Keywords:** population genomics, transcriptomics, gene selection, polydactyly trait, chicken

## Abstract

**Introduction:**

Polydactyly—the presence of extra digits—is a heritable limb anomaly observed in several chicken breeds. The Puan Panjiang black-bone chicken uniquely exhibits both four- and five-toed phenotypes, yet the genetic and transcriptional bases of this trait remain unclear. This study aimed to elucidate the genomic variants and gene expression changes underlying polydactyly in this breed.

**Methods:**

We performed whole-genome resequencing (WGS) on 43 Puan Panjiang chickens (22 four-toed, 21 five-toed) and integrated publicly available data from 17 red junglefowl (RJF). After stringent quality filtering, we aligned reads to GRCg7b, identified high-confidence SNPs and InDels, and conducted sliding-window analyses of nucleotide diversity (θπ) and genetic differentiation (F <sub>ST</sub>) to detect selective sweeps. Concurrently, we carried out RNA-seq on embryonic foot tissues at days 6–9 (24 four-toed, 24 five-toed samples), quantified transcript levels (TPM), and identified differentially expressed genes (DEGs) with DESeq2 (adjusted *P* < 0.01, |log_2_FC|> 2). Fuzzy c-means clustering delineated temporal expression patterns, and enrichment analyses (KEGG, GO) characterized candidate pathways.

**Results:**

Genomic scans revealed 1,339 and 1,035 positively selected genes in five-toed and four-toed chickens, respectively, with 335 shared loci relative to RJF. Top candidates in polydactylous birds included AUH, SEMA4D, and ROR2, while four-toed birds showed strong signals at RYR2, KITLG, and PGR. KEGG enrichment highlighted the MAPK signaling pathway in both groups, and uniquely in five-toed birds, lipid metabolism and vascular signaling pathways (e.g., sphingolipid and apelin signaling). Transcriptome profiling demonstrated that the greatest transcriptional divergence between phenotypes occurred at embryonic Days 8–9, pinpointing a critical window for extra-digit differentiation. Clustering analyses indicated coordinated regulation of genes involved in ribosome biogenesis, extracellular matrix organization, and muscle development across stages.

**Discussion:**

Our integrated analyses pinpoint MAPK pathway genes and lipid-vascular interactions as central to extra-toe formation, with the Days 8–9 embryonic window being pivotal. These findings offer clear targets for functional validation and may guide selective breeding for limb traits in poultry.

## 1 Introduction

Since the 17th century, the occurrence of supernumerary fingers or toes in humans and other quadrupeds has attracted widespread interest and subsequently had a profound impact on scientific research on developmental biology, biogenetics, and evolutionary theory (1, 2). Polydactyly is a common hereditary limb deformity in many vertebrates (3). Clinically, polydactyly is caused by defective patterning of the anterior–posterior axis of the developing limbs, manifesting as an isolated disease or as part of an abnormal syndrome (4). Several genes have been implicated in human polydactyly, including GLI3, ZNF141, MIPOL1, and PITX1 (5).

In chickens, polydactyly manifests as extra toes on one or both feet and occurs in specific breeds such as Silkie, Beijing Fatty, and Dorking chickens (6). Previous studies have located the Po gene in chickens on chromosome 2 and focused on the role of the LMBR1 gene in the development of polydactyly in Silky chickens and Beijing Fatty Chickens (7–9). During the early stages of limb development, ectopic expression of several genes, including SHH and FGF4, was found in the Dorking chicken hindlimb, of which FGF4 was upregulated (10). The application of the SHH protein to the anterior limb of developing chick embryos induces the formation of ectopic digits in a concentration- and time-dependent manner. Furthermore, in mice and chickens, the loss of SHH expression results in the absence of digit bones (11–13).

The Puan Panjiang black-bone chicken is one of the rarest poultry in China (14). It is famous for its black feathers, black legs, black skin, black meat, black bones, strong resistance to adversity, and delicious meat. This breed of chicken has both four-toed and five-toed individuals, and this polydactyly makes the Puan black-bone chicken even more unique. However, although this polydactyly trait is relatively common in this breed, the genetic background of polydactyly in the Puan Panjiang black-bone chicken has not been fully studied and elucidated. In addition, owing to its morphological changes, the Puan Panjiang black-bone chicken can be an ideal model for studying polydactyly and related phenotypes in poultry.

With the rapid development of genomic technologies, especially the application of whole genome resequencing (WGS) and transcriptome sequencing (RNA-seq), we can identify genetic variants and candidate genes associated with polydactyly at the genome and transcriptome scales (15–17). These technological advances have provided new tools and methods for studying polydactyly in chickens, helping us to gain a deeper understanding of the genetic background of this trait (18, 19). The content of this research has been consistent with all the basic causes, weights, and numbers, and the structure of the system shows a multi-toed structure with a different molecular mechanism. We collected and analyzed the genome and transcriptome data of chickens with polydactyly and normal chickens, to reveal the genes and regulatory networks associated with this trait and provide a reference for future genetic research and poultry breeding.

## 2 Materials and methodology

### 2.1 Animals and sample collection

**Figure 1:**
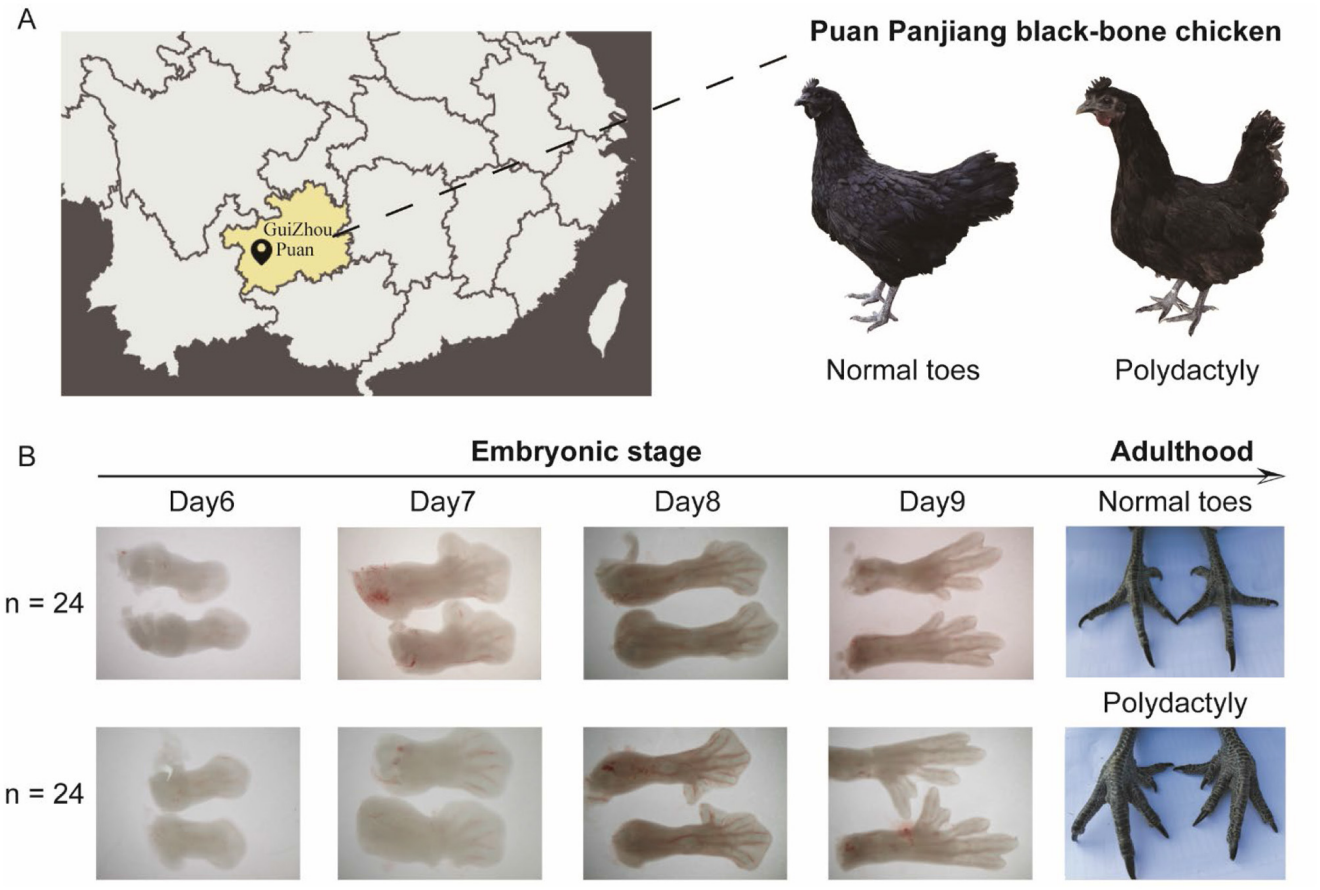
Sampling location and morphological comparison of Puan Panjiang black-bone chickens. **(A)** Geographic location of Puan, GuiZhou, where the Puan Panjiang black-bone chicken samples were collected. **(B)** Morphological differences in foot development between the PPNT and PPP during the embryonic and adult stages.

For whole-genome resequencing, we collected 43 chicken blood samples from the wing vein: 22 from Puan Panjiang black-bone chickens with normal toes (PPNT) and 21 from those with polydactyly (PPP) (Figure 1A, Table 1). Additionally, 17 red jungle fowl (RJF) genome sequences were retrieved from the NCBI (Supplementary Table S1) for comparative genomic analysis. All samples were stored at −20°C. For transcriptome sequencing, we also used 48 Puan Panjiang black-bone chicken embryo samples (24 PPNT and 24 PPP), which were incubated for 6–9 Days (Table 1). The Puan Panjiang black-bone chickens used in this study were provided by Guizhou Jinhe Poultry Co., Ltd. Chickens were reared in a controlled environment under standardized management protocols. The breeding cycle was divided into four stages: brooding (0–6 weeks of age), growing (7–18 weeks of age), pre-laying (19–20 weeks of age), and laying (21–43 weeks of age). During the brooding period (0–6 weeks), chickens were housed at a density of 50 birds/m^2^, with ambient temperature gradually reduced from 32°C to 23°C and photoperiod adjusted from 24 to 9 h/day. In the growing period (7–18 weeks), the density was reduced to 12 birds/m^2^, with temperature maintained at 23°C and photoperiod at 9 h/day. During the laying period (19–43 weeks), chickens were individually caged under a photoperiod of 16 h/day and temperature of 23°C. All chickens had *ad libitum* access to water and a standard commercial diet. Regular disinfection and vaccination protocols were strictly followed. The selection of sample size was based on a priori power analysis, ensuring the detection of subtle genetic, and expression differences within the population at 80% statistical power and a significance level of α = 0.05. This balanced grouping not only met the requirements of the central limit theorem but also robustly captured transcriptional fluctuations during critical developmental stages, thereby guaranteeing the reliability of subsequent analysis results. Significant morphological differences between the groups were observed from the embryonic stage to adulthood (Figure 1B). All eggs were obtained from a controlled experimental chicken farm in Puan County, Guizhou Province, China, and were sampled in the hatching grid at Chengdu University. Specifically, eggs were incubated at 38.5°C with appropriate humidity until the 6–9 Days embryonic stage, ensuring the embryos reached the predetermined developmental stage. The blunt end of each egg (the air chamber end) was then carefully opened with a microscalpel, creating a 1 cm−2 cm incision. Using forceps, the eggshell membrane was gently removed to avoid damage to the embryo. The embryo was then carefully grasped and separated from the amniotic membrane. After removal, the embryos were immediately submerged in phosphate-buffered saline (PBS). Following euthanasia, the embryos were examined under a dissecting microscope, and images were captured. The hind limb area was located, and the toes were carefully excised with a microscalpel, preserving the morphology as much as possible. The toe samples were then frozen in liquid nitrogen and stored at −80°C for subsequent analysis. When euthanizing chicken embryos on Days 6–9 of the embryonic period, carbon dioxide inhalation is used. The embryos are placed in a sealed container and high-purity carbon dioxide is introduced at a volume flow rate of 30%−70% of the container per minute, with an initial concentration of about 70%. During the ventilation process, the embryo's reactions, such as struggling and opening its mouth, are closely observed. Generally, after a few minutes, the embryo will gradually become comatose and its activity will decrease until it completely stops moving and is confirmed dead (judged by observing vital signs such as heartbeat and breathing).

**Table 1:** Summary of genome resequencing and transcriptome sampling schedule.

**Species**	**Genome resequencing (*n*)**	**Transcriptome sequencing (** ***n*** **)**			
		**Day 6**	**Day 7**	**Day 8**	**Day 9**
Puan Panjiang black-bone chicken (PPNT)	22	6	6	6	6
Puan Panjiang black-bone chicken (PPP)	21	6	6	6	6
Red jungle fowl (RJF)	17	NA	NA	NA	NA

### 2.2 Genomic DNA extraction

Total DNA was isolated from blood using the TIANamp Genomic DNA Kit (Tiangen Biotech) according to the manufacturer's instructions. The extracted DNA concentration and purity were assessed using a NanoDrop2000 spectrophotometer (Thermo Fisher Scientific, Chengdu, China), and its integrity was assessed by 1% agarose gel electrophoresis.

### 2.3 Whole-genome sequencing and quality control

After verifying the quality of the DNA samples, DNA was fragmented randomly using an ultrasonicator (Covaris Inc., Woburn, MA, USA). A sequencing library was then prepared with the NEBNext^®^ Ultra™ DNA Library Prep Kit (Illumina, NEB, USA, Catalog #: E7370L) according to the manufacturer's instructions. Indexing was performed to uniquely tag each sample. The library preparation process involved end repair, dA tailing, and ligation with a full-length adapter for sequencing, followed by PCR amplification and purification. The resulting DNA libraries, with an insert size of 350 bp, were constructed. Sequencing of the genomes of 43 individuals was performed using 150 bp paired-end reads on the DNBSEQ-T7 platform (Novogene Bioinformatics Technology Co., Ltd., Beijing, China). To minimize sequencing errors and reduce noise in the analysis, low-quality paired reads were discarded based on the following criteria: (i) ≥10% unidentified nucleotides [N], (ii) >10 nt aligned to the adapter (allowing ≤ 10% mismatches), (iii) >50% bases with Phred quality < 5, and (iv) putative PCR duplicates generated during library construction. An in-house script was used for this quality control step. As a result, 0.98 terabases (~21.69-fold coverage per individual) of high-quality paired-end reads were obtained, with 95.83% of nucleotides having a Phred quality ≥ Q30 (≥98.83% base call accuracy).

The remaining high-quality reads were aligned to the reference chicken genome (GRCg7b) using the Burrows-Wheeler Alignment tool (BWA, version 0.7.15) with the command “mem -t 10 -k 32”. SAMtools (v0.1.19) was used to convert SAM to BAM and perform initial sorting (20). Reads with ≤ 5 mismatches and mapping quality score >40 were retained to ensure alignment precision. After initial sorting with SAMtools, duplicate reads were marked using the “MarkDuplicates” command from the Picard package (version 1.119).

Variant calling was performed using the Genome Analysis Toolkit (GATK) best practices pipeline (version 3.7) (21), employing the HaplotypeCaller method. Population SNP (Single Nucleotide Polymorphism) calling was conducted by merging all generated gVCFs using the “CombineGVCFs” command. To ensure the reliability of identified SNPs, stringent filtering criteria were applied during the variant filtration step: (a) quality by depth > 10.0, (b) mapping quality score > 40.0, (c) FS < 60.0, (d) MQRank-Sum > −12.5, (e) ReadPosRankSum > −8.0. Additionally, SNPs with inter-site distances ≤ 5 were excluded to minimize linkage artifacts (22). For the final selection of high-confidence SNPs, we used vcftools (version 0.1.15) with the following parameters: sample call rate > 90%, SNP calls rate > 95%, minor allele frequencies > 1%, and Hardy-Weinberg equilibrium *P*-value < 10^−^5. After stringent filtering, 11.65 million high-confidence SNPs were identified in PPNT and 14.50 million in PPP. These SNPs were categorized into various genomic regions, including exonic, intronic, splice site, upstream and downstream gene regions, and intergenic regions, and annotated using the ANNOVAR package (23).

### 2.4 Calculation of θπ and F_*ST*_

A sliding-window approach (40-kb windows sliding in 10-kb steps) was used to quantify polymorphism levels (θπ, pairwise nucleotide variation as a measure of variability) (24) and genetic differentiation (*Fst*) (25) between RJFs and two chmsicken breeds. Based on this, we further combined θπ with the *Fst* statistic and implemented a selective sweep screening method to identify genomic regions that may be affected by natural selection in different populations of Puan Panjiang Black-bone Chicken.

### 2.5 Total RNA-seq and data analysis

Total RNA was extracted from tissue samples (pedal region) using the Qiagen RNeasy Kit. Sequencing libraries for 48 samples were prepared with the NEBNext Ultra RNA Library Prep Kit for Illumina (NEB, USA, Catalog #: E7530L) following the manufacturer's instructions. mRNA was isolated using poly-T oligo-attached magnetic beads. Paired-end sequencing (2 bp × 150 bp) was performed on the DNBSEQ-T7 platform. The high-quality reads obtained were mapped to the chicken reference genome using TopHat2 (26). Aligned reads were assembled with StringTie v1.3.3, and transcript construction was completed using Cufflinks 2.0.2 (27, 28). Transcript expression levels were quantified as transcripts per million (TPM) values using StringTie. Transcripts with a TPM ≥ 0.5 in at least two biological replicates were considered as expressed protein-coding genes (PCGs). Differential expression analysis of PCGs was performed using DESeq2 (29). Genes were considered statistically significant when their adjusted *P*-value was < 0.01 and their absolute log_2_ fold change was >2. To explore the biological significance of differentially expressed genes, gene ontology enrichment analysis for each cluster was conducted using Metascape (30, 31). In RNA-seq data analysis, we use the Mfuzz package to implement fuzzy c-means clustering to study the expression dynamics of genes at different time points. The specific steps are as follows: first, after constructing the ExpressionSet object, use the filter.NA function (threshold 0.25) to remove genes with more missing data, and use fill.NA (mode = “mean”) to fill the missing values. Then call filter.std to filter out genes with low standard deviations and standardize the data; then, perform clustering analysis by setting the number of clusters *c* = 6 and using the mestimate function to automatically estimate the most appropriate fuzzy index m (this parameter determines the fuzziness of the clustering), and set the random seed set.seed (150) to ensure the reproducibility of the results. Finally, use the mfuzz.plot2 function to visualize the clustering results by time point (with the all_TPM_matrix column name as the label).

## 3 Results

### 3.1 Sequencing and variation discovery

We sequenced 43 Puan Panjiang black-bone chickens from Puan County, Guizhou Province, and performed transcriptome sequencing on 48 (24 four-toed and 24 five-toed) 6–9 Day-old Puan Panjiang black-bone chicken embryonic foot tissues (Figure 1B), obtaining ~0.98 trillion bases (Tb) of genome resequencing data. After screening, we aligned the reads to the reference chicken genome, achieving coverage of ~21.69 times per individual (Supplementary Table S1). In addition, we also included previously published genome sequence data of 17 red junglefowl (RJF), with an average coverage of ~23 times per individual, which were obtained from the downloaded and analyzed datasets (GenBank accession numbers are provided in Supplementary Table S1; Table 1). After this large dataset was aligned with the reference chicken genome, we identified ~11.65 million and 14.5 million single-nucleotide polymorphisms (SNPs) and 13.51 million and 15.57 million insertions and deletions (InDels) in PPNT and PPP, respectively (Table 2). To comprehensively explore the mRNA expression profile of the foot tissue of the PPNT and PPP at different time points, we constructed a total of 48 cDNA libraries. The dataset included 324.66 gigabases (Gb) of clean data, with an average of 6.76 per sample (Supplementary Table S2).

**Table 2:** SNP and InDels categories in two chicken breeds.

**SNP Category**			**PPNT**		**PPP**	
			**Number**	**Ratio**	**Number**	**Ratio**
Upstream			174,400	1.50%	216,527	1.49%
Gene body	CDS	Synonymous	110,843	0.95%	139,275	0.96%
		Nonsyn/Syn ratio (ω)	63,290	0.54%	79,364	0.55%
		Stop gain	771	0.01%	997	0.01%
		Stop loss	103	0.00%	117	0.00%
		unknown	27	0.00%	39	0.00%
	Intronic		4,964,981	42.62%	6,175,900	42.60%
	Splicing		407	0.00%	501	0.00%
Downstream			152,505	1.31%	189,824	1.31%
Upstream/Downstream			10,058	0.09%	12,554	0.09%
Intergenic			5,998,174	51.48%	7,463,411	51.48%
Exonic			175,022	1.50%	219,777	1.52%
Exonic/Splicing			12	0.00%	15	0.00%
Total			11,650,593	100.00%	14,498,301	100.00%
**InDels category**						
Upstream			20,667	1.53%	23,713	1.52%
Gene body	CDS	Frameshift deletion	1,078	0.08%	1,267	0.08%
		Frameshift insertion	1,202	0.09%	1,311	0.08%
		Frameshift substitution	320	0.02%	346	0.02%
		Non-frameshift deletion	745	0.06%	880	0.06%
		Non-frameshift insertion	512	0.04%	579	0.04%
		Non-frameshift substitution	172	0.01%	188	0.01%
		Stopgain	12	0.00%	13	0.00%
		Stoploss	7	0.00%	8	0.00%
	Intronic		589,368	43.63%	681,902	43.79%
	Splicing		165	0.01%	182	0.01%
Downstream			21,895	1.62%	24,902	1.60%
Upstream/Downstream			1,483	0.11%	1,687	0.11%
Intergenic			713,303	52.80%	820,163	52.67%
Total			1,350,930	100.00%	1,557,142	100.00%

### 3.2 Genome-wide selective sweep signals in PPP and PPNT

**Figure 2:**
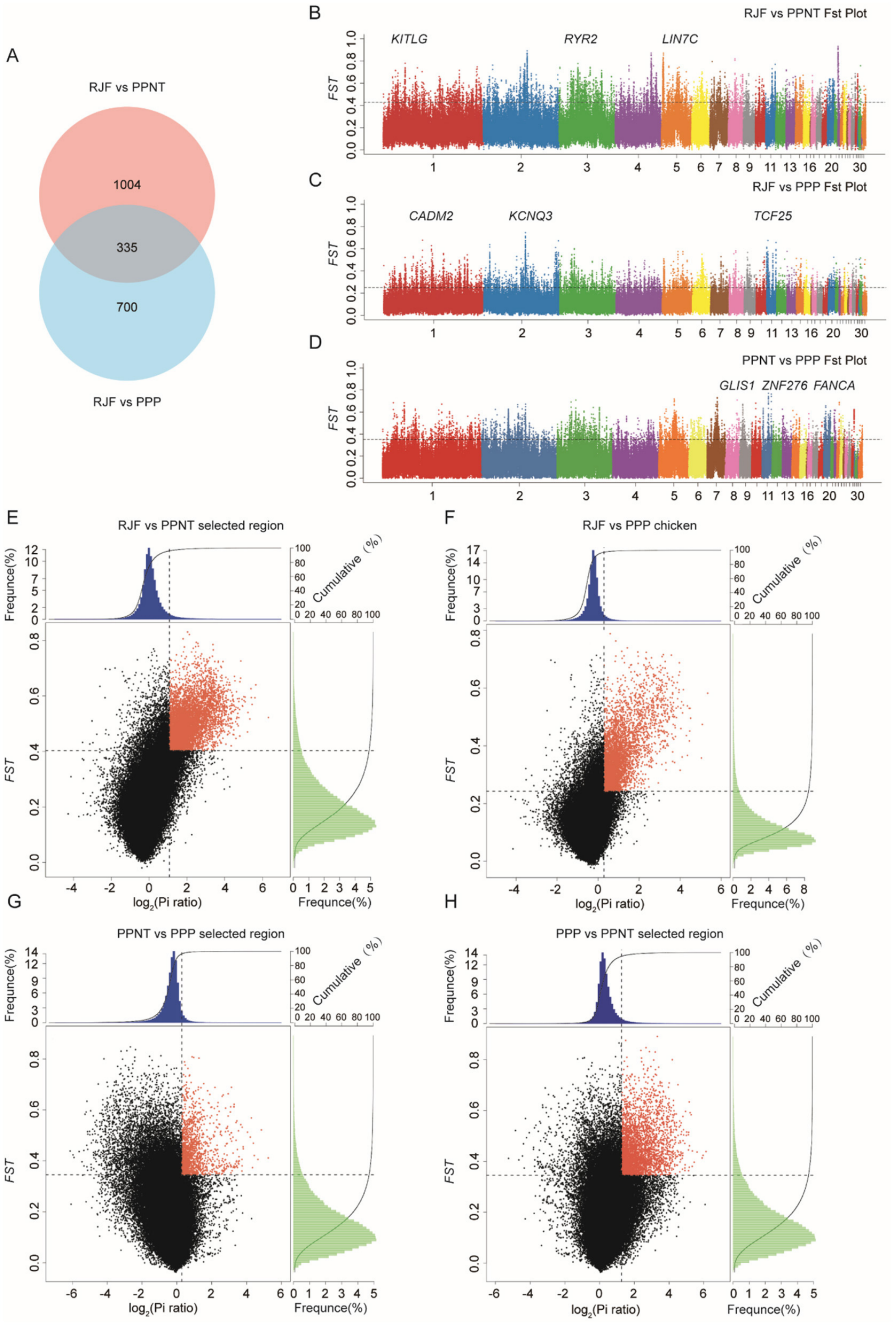
Genomic differentiation and selective sweeps among RJF, PPNT, and PPP. **(A)** Venn diagram showing the number of selected genes identified by comparing RJF vs. PPNT and RJF vs. PPP. **(B–D)** Manhattan plots of *Fst* values across the genome for different pairwise comparisons: RJF vs. PPNT **(B)**, RJF vs. PPP **(C)**, and PPNT vs. PPP **(D)**. The Y-axis represents the *Fst* values, and the X-axis represents the genomic positions across 30 chromosomes. Significant peaks indicate regions of high genetic differentiation, with key candidate genes labeled. **(E–H)** Scatter plots of *Fst* vs. the log2(Pi ratio) ratio for RJF vs. PPNT **(E)**, RJF vs. PPP **(F)**, PPNT vs. PPP **(G)**, and PPP vs. PPNT **(H)**. Red dots indicate genomic regions under strong selection, characterized by high *Fst* values and significant differences in nucleotide diversity (Pi ratio). The frequency distributions of *Fst* and the Pi ratio are shown in the top and right histograms of each plot.

Through analysis, we identified 1,035 and 1,339 positively selected genes (PSGs) in the PPNT and PPP, respectively (Figure 2A). This indicates that there are strong selective sweep signals in both species. There are 335 common genes with strong selective clearance signals when the two populations are compared with RJFs. Compared with RJF, the regions of interest selected from PPNT (Figure 2B) and PPP (Figure 2C) presented significantly greater *Fst* values (within the top 5%). Similarly, compared with those of PPNT, the regions of interest selected from PPP (Figure 2D) presented elevated *Fst* values (within the top 5%). These Manhattan plots highlight the genetic differentiation of the genome between chicken populations, with specific loci showing clear differentiation. Regions showing significant differences (*p* < 10–16, Mann-Whitney *U*-test) in the log2 (θπ ratio) and *Fst* values compared with the wild RJF genomic background are highlighted for both the PPNT (Figure 2E) and PPP (Figure 2F) chicken populations. When the PPNT and PPP were compared, regions showing significant differences were highlighted in the PPP (Figure 2G) and PPNT (Figure 2H) chicken populations.

Notably, compared with the wild-type RJF genomic background, 75 regions and 617 regions presented *Fst* values that surpassed 0.5 for PPP and PPNT, respectively. The top 10 selected genes according to *Fst* size are shown in Table 3. Within these selected regions in the PPP, 53 known genes were identified, including AUH, SEMA4D, NA, NXNL2, ROR2, KDM4C, PTCH1, FANCC, ANKRD55, and GPBP1. Similarly, PPNT revealed 406 known genes, including RYR2, TPGS2, NA, ZNF608, KITLG, KIAA1328, PGR, LIN7C, NADK2 and ACTN2, within its selected regions.

**Table 3:** Top 10 selected genes in RJF compared to two chicken populations.

**RJF vs. PPP**		**RJF vs. PPNT**	
**Gene**	***Fst***	**Gene**	***Fst***
AUH	0.724758	RYR2	0.777158
SEMA4D	0.723573	TPGS2	0.772454
NA	0.710409	NA	0.772454
NXNL2	0.703419	ZNF608	0.742762
ROR2	0.692182	KITLG	0.734120
KDM4C	0.685189	KIAA1328	0.733631
PTCH1	0.680427	PGR	0.725993
FANCC	0.678521	LIN7C	0.721228
ANKRD55	0.674275	NADK2	0.716742
GPBP1	0.663810	ACTN2	0.710283

### 3.3 Selection signatures for PPP and PPNT

**Figure 3:**
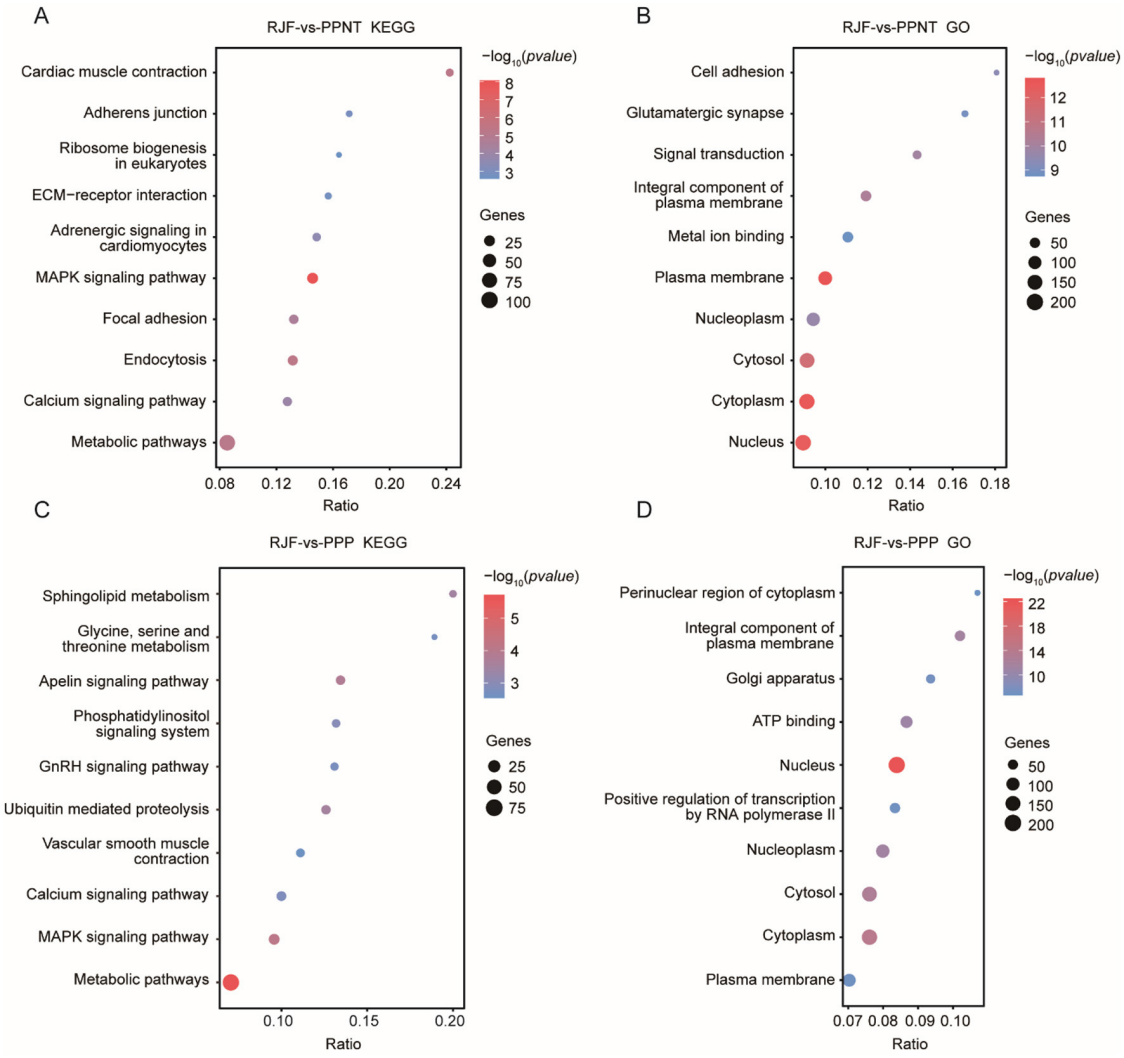
KEGG and GO enrichment analysis of the two chicken populations compared with RJF. The dot plots show the results of the KEGG **(A, C)** and GO **(B, D)** enrichment analyses of selected genes in the PPNT and PPP with RJF. The color indicates the *P*-value, and the circle indicates the gene count.

KEGG analysis revealed that the pathways enriched in the comparison between PPNT and RJF mainly included pathways related to cell adhesion, signal transduction, and metabolism. The notable pathways included cardiac muscle contraction, adherens junction, ECM-receptor interaction, the MAPK signaling pathway, focal adhesion, and the calcium signaling pathway. Among these pathways, the MAPK signaling pathway exhibited a high enrichment ratio, indicating its potential role in the physiological differences between RJF and PPNT. Additionally, ribosome biogenesis in eukaryotes and metabolic pathways were significantly enriched, highlighting processes linked to protein synthesis and fundamental cellular metabolism. In contrast, the comparison between PPP and RJF revealed several other enriched pathways. These included sphingolipid metabolism; glycine, serine and threonine metabolism; the apelin signaling pathway; and the phosphatidylinositol signaling system. Pathways associated with hormonal and vascular regulation, such as the GnRH signaling pathway and vascular smooth muscle contraction, were also significantly enriched. Unlike the former, sphingolipid metabolism and the apelin signaling pathway are unique to the PPP, suggesting potential differences in lipid metabolism and vascular signaling mechanisms (Figures 3A, C).

The GO terms enriched in the comparison between PPNT and RF included the nucleus, cytosol, and plasma membrane (Figure 3B). Compared with the former, the GO term analysis of the PPP revealed that it was related to subcellular compartments and transcriptional regulation, and it was enriched in the perinuclear region of the cytoplasm, Golgi apparatus, and ATP binding (Figure 3D). Notably, positive regulation of transcription by RNA polymerase II was highly enriched, suggesting an adaptive emphasis on transcriptional control in the PPP.

### 3.4 Overview of transcriptome dynamics during toe development in two chicken populations

**Figure 4:**
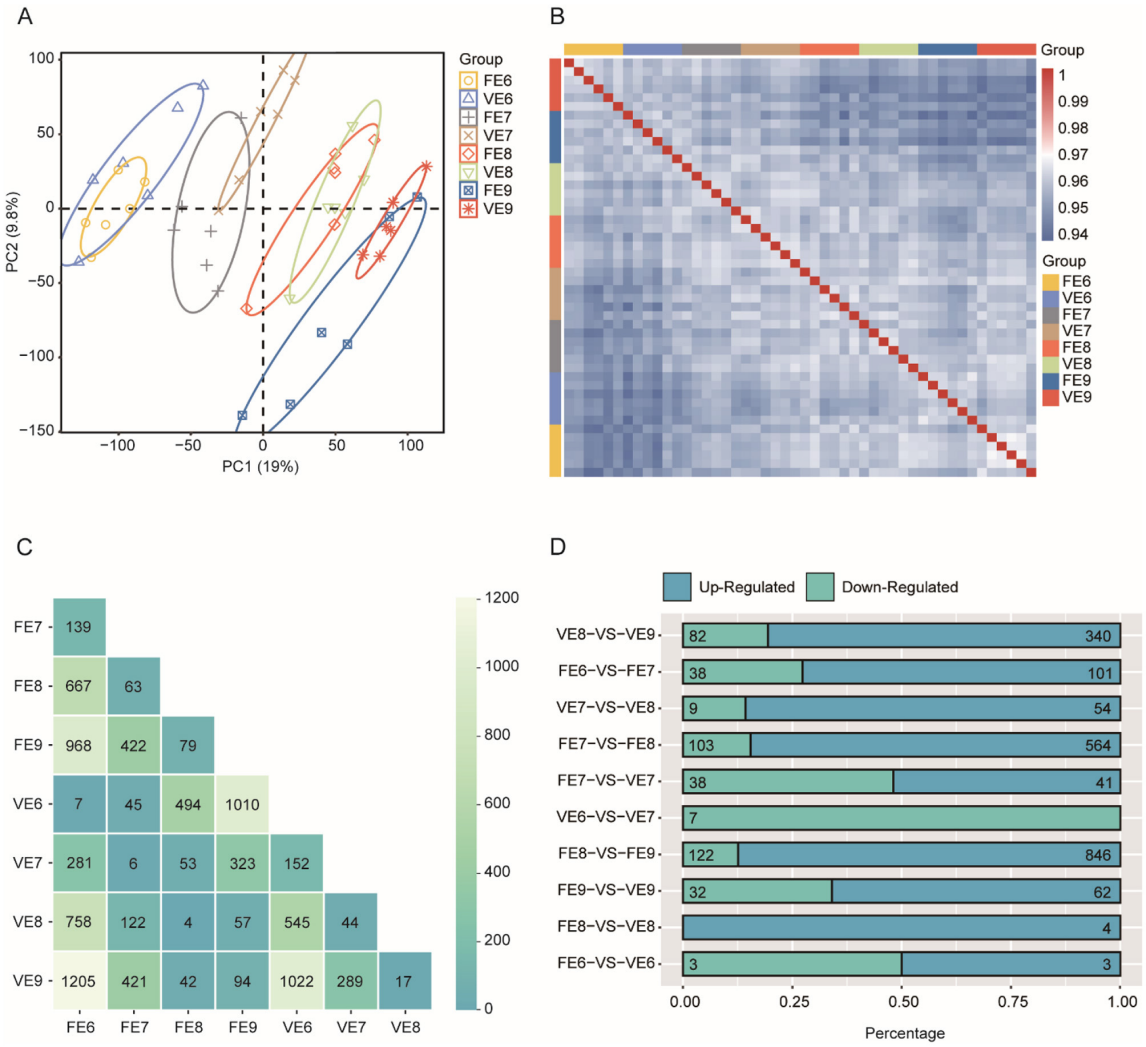
Overview of transcriptomes of two chicken populations of Panjiang black-bone chicken. **(A)** PCA based on TPM values of genes in RNA-seq samples. FE refers to four-toed chicken embryo (FE), VE refers to five-toed chicken embryo (VE), and 6, 7, 8, and 9 refer to the number of days for the egg to hatch. **(B)** Spearman correlation heatmap showing the correlation between each RNA-seq sample. **(C)** Heatmap of the number of pairwise comparisons of DEGs in toe samples from two chicken populations at four time points. **(D)** Bar graph showing the proportion of differentially upregulated (green) and downregulated (blue) genes to the total number of DEGs in toe samples from four-toed and five-toed chickens from the same day and from two adjacent periods of the same chicken.

The PCA results revealed that the samples were clustered according to different developmental stages and presented a temporally continuous distribution from embryonic stages D6–D9 (Figure 4A). Greater correlations were found among the 6 replicate samples from the same developmental stage (Figure 4B). We then performed a 2 × 2 differential expression analysis for the four developmental stages and calculated the number of DEGs with significant differences in each control group (adjusted *P*-value < 0.01 and absolute value of log_2_FoldChange > 2; Figure 4C). In the same chicken population, the fewest genes were differentially expressed between two adjacent developmental stages, and as development progressed, the number of genes that were differentially expressed between stages gradually increased. Notably, the number of DEGs associated with the PPNT and PPP were the lowest during the same time period but increased on the ninth day (*N* = 94; Figure 4D), indicating that the toe transcriptomes of the PPNT and PPP occurred at this stage. There were great changes, and the development process from the embryonic stage D8–D9 is a key turning point in the differentiation of chicken toes.

### 3.5 Dynamic expression landscape of genes in FE and VE

**Figure 5:**
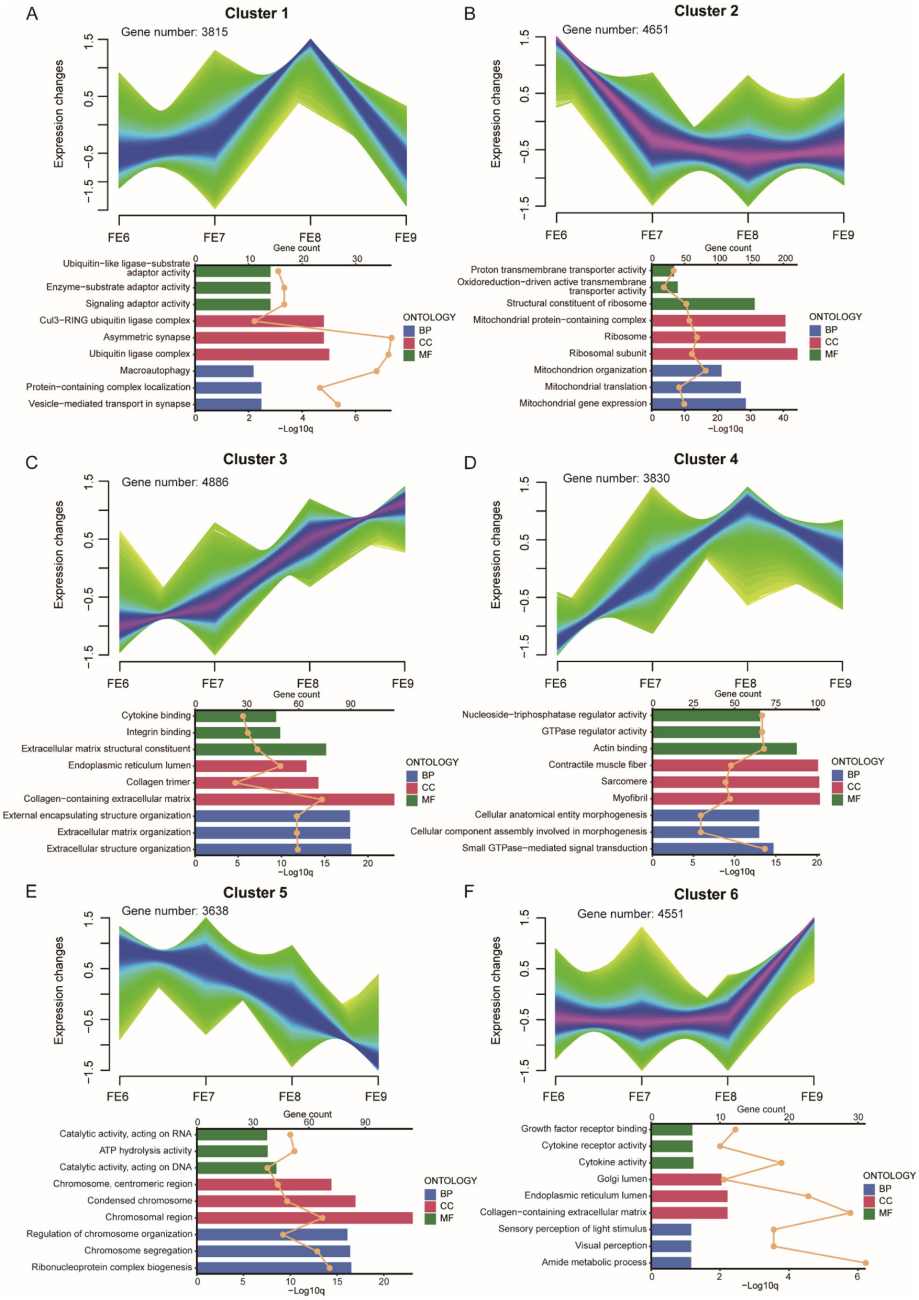
FE dynamic gene expression landscape. **(A–F)** Clustering of fuzzy c-means for six different temporal patterns of gene expression. The bar chart below shows the GO terms for each cluster. Different colors represent the three types of GO terms: biological processes (blue), cellular components (red), and molecular functions (green). The upper axis corresponds to the line graph, which represents gene numbers, and the lower axis corresponds to the bar graph, which represents *P*-value.

**Figure 6:**
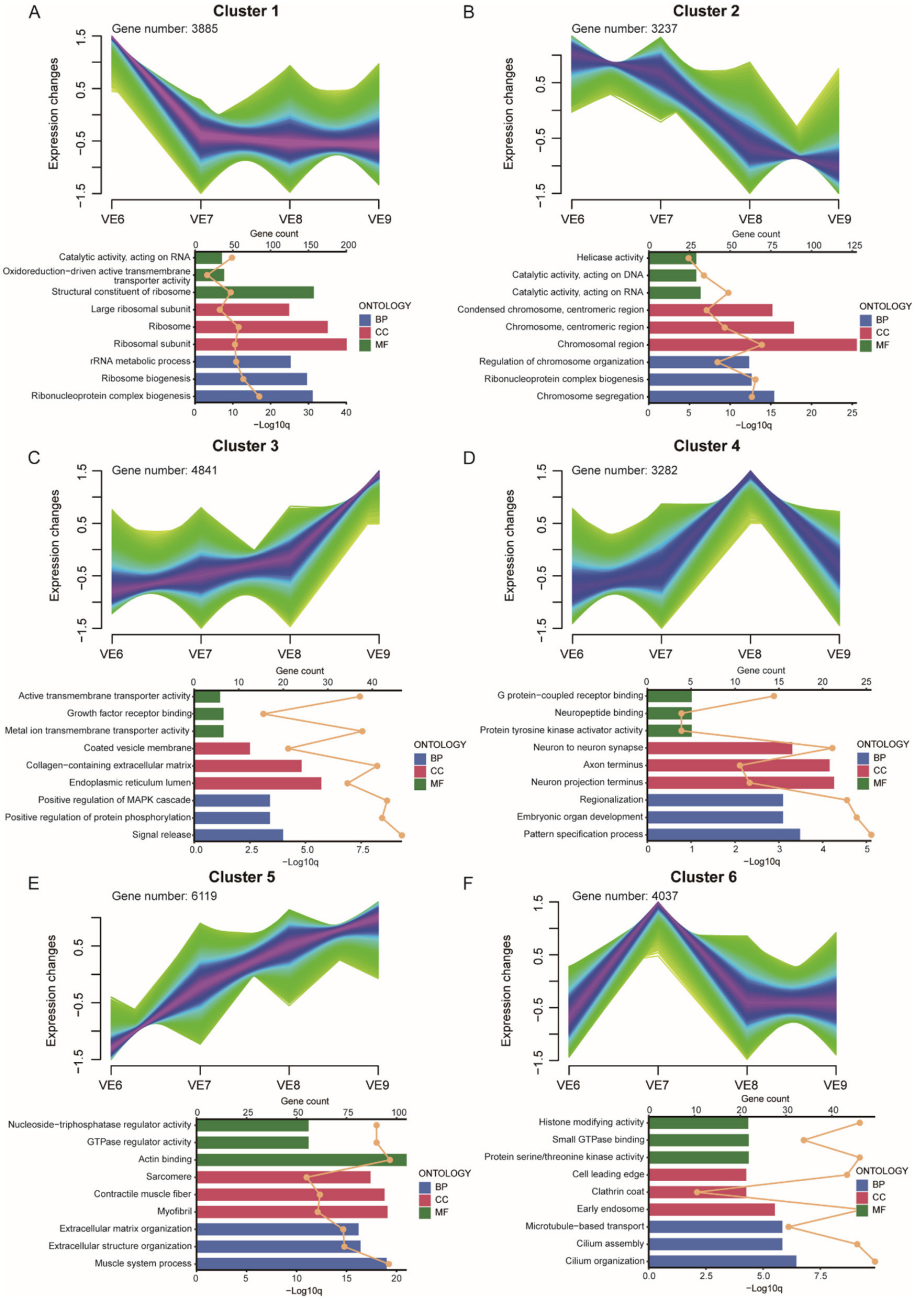
VE dynamic gene expression landscape. **(A–F)** Clustering of fuzzy c-means for 6 different temporal patterns of gene expression. The bar chart below shows the GO terms for each cluster. Different colors represent the three types of GO terms: biological processes (blue), cellular components (red), and molecular functions (green). The upper axis corresponds to the line graph, which represents gene numbers, and the lower axis corresponds to the bar graph, which represents *P*-value.

We observed that the toes of the PPNT and PPP continuously changed from embryonic Days 6–9 (Figure 1B). Obvious toe differentiation occurred from Days 8–9. To study transcriptome dynamics during toe development, we applied the fuzzy c-means algorithm to cluster gene expression profiles into four stages. The regulatory genes of FE and VE at different stages presented six temporal clustering patterns (Figures 5, 6). In FE6, Gene Ontology (GO) analysis revealed that highly expressed genes in Cluster 2 were associated primarily with terms such as structural constituents of ribosomes, mitochondrial protein-containing complexes, ribosomes, and ribosomal subunits (Figure 5B). By FE7, highly expressed genes in Cluster 5 were enriched in terms such as chromosomes, centromeric regions, condensed chromosomes, chromosomal regions, regulation of chromosome organization, chromosome segregation, and ribonucleoprotein complex biogenesis (Figure 5E). At FE8, highly expressed genes in Cluster 1 were predominantly linked to the Cu13-RiNG ubiquitin ligase complex, asymmetric synapse, and ubiquitin ligase complex (Figure 5A). Concurrently, Cluster 4 genes were associated with actin binding, contractile muscle fibers, sarcomeres, and myofibrils (Figure 5D). The genes in Clusters 1 and 4 presented similar expression patterns, with gradual increases in expression during the early stages, peaking at FE8, and subsequently decreasing. Conversely, genes in Clusters 3 and 6 presented distinct expression patterns, but both peaked at FE9. The Cluster 3 genes were associated primarily with the collagen-containing extracellular matrix, external encapsulating structure organization, extracellular matrix organization, and extracellular structure organization (Figure 5C). In contrast, Cluster 6 genes were enriched in terms such as the Golgi lumen, endoplasmic reticulum lumen, and collagen-containing extracellular matrix (Figure 5F).

In VE6, Gene Ontology (GO) analysis revealed that the highly expressed genes in Cluster 1 were predominantly associated with terms such as structural constituents of ribosomes, ribosomes, ribosomal subunits, ribosome biogenesis, and ribonucleoprotein complex biogenesis (Figure 6A). These genes were highly expressed exclusively in VE6. In VE7, genes enriched in Cluster 6 were associated with terms including cilium organization, cilium assembly, microtubule-based transport, early endosome, protein serine/threonine kinase activity, and histone-modifying activity and were exclusively expressed in this cluster (Figure 6F). In VE8, genes in Cluster 4 were associated primarily with terms such as neuron-to-neuron synapses, axon termini, neuron projection termini, embryonic organ development, and pattern specification processes (Figure 6D). The gene expression patterns in Clusters 3 and 5 were similar; both showed low expression levels early on, which peaked at VE9. The highly expressed genes in Cluster 3 were associated mainly with terms such as the endoplasmic reticulum lumen and collagen-containing extracellular matrix (Figure 6C), whereas the genes in Cluster 5 were related to terms such as the actin binding, sarcomere, and muscle system processes (Figure 6E). The gene expression levels in Cluster 2 gradually decreased over time and were enriched predominantly in terms such as chromosomal regions and chromosome segregation (Figure 6B). For VE6 and VE7, genes involved in ribosome structure, cilium organization, and histone modification were enriched, suggesting that early-stage processes are involved in the development of the extra toe in VE. In contrast, genes associated with neural development, such as those involved in synapse formation, axon development, and neuron projection (VE8), could be important for the differentiation of tissues and organs associated with the extra toe. At VE9, genes related to actin binding, sarcomere, and muscle development (Cluster 5) are highly expressed, which could contribute to the formation and growth of additional toes.

In summary, the results indicate that there are distinct gene expression profiles between normal and polydactylous chicken embryos, especially in relation to the formation of additional toes in PPP chickens. These findings suggest that the regulatory mechanisms controlling chromosome organization, extracellular matrix formation, and muscle development may differ between the two groups, contributing to the extra toe phenotype in PPP chickens.

## 4 Discussion

Polydactyly, characterized by the presence of extra toes, has long been a subject of interest in animal development and genetics (32, 33). In general, chickens have four toes on each foot, but some breeds have more toes, such as Beijing-You, Silkie, Jiningbairi, Dorking, and Houdan (18, 34, 35). However, current studies have not explored the differences in transcriptional changes in the foot bones and skin during development between normal and polydactylous chickens. In this study, we explored the genetic basis of polydactyly in Puan Panjiang black-bone chickens, focusing on the genom e-wide variation and transcriptome dynamics associated with this genetically inherited limb anomaly. Our results provide new insights into the genetic basis of polydactyly in chickens, highlighting the key genes, pathways, and regulatory networks that contribute to this trait.

In this study, the top 10 genes with the highest selection levels in the PPP included *AUH, SEMA4D, NA, NXNL2, ROR2, KDM4C, PTCH1, FANCC, ANKRD55*, and *GPBP1*. The top 10 genes with the highest selection levels identified in PPNT included *RYR2, TPGS2, NA, ZNF608, KITLG, KIAA1328, PGR, LIN7C, NADK2*, and *ACTN2*. The *AUH* gene encodes a bifunctional mitochondrial protein with both RNA binding and hydratase activities. Reduced or overexpressed expression of the protein encoded by *AUH* in cells results in defective mitochondrial translation, which leads to changes in mitochondrial morphology and reduced mitochondrial RNA stability, biogenesis, and respiratory function (36). The protein encoded by the *ROR2* gene is a receptor protein tyrosine kinase and type I transmembrane protein that belongs to the *ROR* subfamily of cell surface receptors. The protein may be involved in the early formation of chondrocytes and may be required for the development of cartilage and growth plates (37). Mutations in this gene can cause brachydactyly type B, a skeletal disorder characterized by hypoplasia/hypoplasia of the distal phalanges and nails (38–40). In addition, mutations in this gene can cause an autosomal recessive form of Robinow syndrome, characterized by skeletal dysplasia with generalized shortening of limb bones, segmental defects of the spine, brachydactyly, and a dysmorphic facial appearance (38, 41). Therefore, the selection signal of this gene observed in polydactyl chicken species in this study may reflect its role in the formation of abnormal limb patterning. *KITLG* (KIT ligand) is a protein-coding gene, and its related pathways include apoptosis pathways and GPCR pathways in synovial fibroblasts. *PRG4* synovial fibroblasts secrete R-spondin-2 to promote the occurrence and development of osteoarthritis (42). We speculate that these genes may affect the development of polydactyly in chickens; however, their exact mechanisms of action remain unclear.

In addition to the identification of candidate genes, we conducted a comprehensive analysis of transcriptome dynamics during toe development in normal and polydactylous chickens. Our results revealed that the greatest changes in gene expression occurred between embryonic Days 8 and 9, suggesting that this period represents a critical window for toe differentiation. In human studies on polydactyly, the overall detection rate of polydactyly by prenatal ultrasound is 19.2%, and the first detection rates in early, middle and late pregnancy are 0.9%, 14.6%, and 3.7%, respectively (43, 44). This phenomenon, namely, the high incidence of polydactyly in fetuses during the second trimester, is similar to the peak gene expression observed in the present study starting on the ninth day of embryonic development.

Furthermore, pathway enrichment analysis revealed that several biological pathways were significantly associated with polydactyly in Puan Panjiang black-bone chickens. Compared with that in wild-type red junglefowl (RJF), the MAPK signaling pathway, which is involved in cell differentiation, growth, and survival, was highly enriched in both PPP and PPNT. Zhang et al. (45) emphasized the important role of the MAPK family in complex cellular programs such as proliferation, differentiation, development, transformation, and apoptosis. These findings suggest that the MAPK pathway may be a key regulator of limb development in chickens. In contrast, PPP chickens were significantly enriched in pathways related to lipid metabolism and vascular signaling, such as sphingolipid metabolism and the apelin signaling pathway, which were not enriched in PPNT chickens. Sphingolipids have been implicated in the regulation of cell growth, differentiation, and programmed cell death. The current paradigm for their role is that complex sphingolipids interact with growth factor receptors, the extracellular matrix, and neighboring cells (46, 47). In addition, increasing evidence indicates that apelin is involved in the regulation of skeletal muscle metabolism. Dray et al. demonstrated that acute apelin treatment can increase the rate of skeletal muscle glucose disposal and that long-term treatment leads to weight gain and reduced fat pad mass in mice while increasing the expression of uncoupling protein 3 (UCP3) in mouse skeletal muscle (48–50). These findings indicate that the occurrence of polydactyly is related to certain neural regulatory mechanisms. This difference in pathway enrichment may reflect the distinct physiological processes that support the formation of additional toes in PPP chickens, potentially involving changes in lipid metabolism, cell signaling, and vascular development.

With the help of integrated analysis, we found that the candidate genes not only showed significant differences in genomic screening, but also showed an expression peak associated with the key embryonic stage (D8–D9) in the dynamic changes of the transcriptome. This stage is the critical period for the differentiation of chicken embryo foot tissue. In particular, the expression changes of genes involved in the MAPK signaling pathway, cell proliferation and apoptosis, cytoskeleton remodeling, and lipid metabolism overlapped with the regions in the genome that showed strong selective signals. It can be inferred that genetic polymorphisms and variations in cis-regulatory elements may affect transcription factor binding, thereby regulating the activity of these pathways, and ultimately affecting the development of specific limb structures (such as the formation of extra toes).

It should be pointed out that this study still has certain limitations. Due to the relatively small sample size, especially in whole genome sequencing, the detection, and statistical power of some low-frequency variants may be affected. In addition, the subjects of this study are mainly a single chicken species, and the universality of the results still needs to be verified in more breeds. Future studies can further enhance the depth and wide applicability of the research by expanding the sample size, introducing multi-breed controls, and conducting functional verification experiments.

In summary, this study provides valuable insights into the genetic and molecular mechanisms underlying polydactyly in Puan Panjiang black-bone chickens. By integrating genomic, transcriptomic, and functional analyses, we identified key genes and pathways that may be involved in the development of additional toes. These findings not only enhance our understanding of polydactyly in chickens but also offer a foundation for future studies aimed at elucidating the broader genetic and developmental mechanisms of limb malformations in vertebrates. Given the unique characteristics of Puan Panjiang black-bone chickens, including their resistance to adversity and economic value, further research into the molecular basis of polydactyly in this breed could have significant implications for poultry breeding and genetic improvement strategies.

## 5 Conclusion

This study provides a comprehensive analysis of the genetic and transcriptomic basis of polydactyly in Puan Panjiang black-bone chickens, identifying key candidate genes and pathways involved in the development of extra toes. Our findings highlight significant differences in gene expression and genomic variation between normal and polydactylous chickens, with the AUH, SEMA4D, and ROR2 genes emerging as key candidates. Pathway enrichment analysis further revealed that the MAPK signaling pathway plays a central role in limb development, whereas lipid metabolism and vascular signaling pathways are uniquely associated with polydactylous chickens. Transcriptome dynamics during embryonic development underscore the critical role of Days 8–9 in the differentiation of the extra toe phenotype. These results not only enhance our understanding of polydactyly in chickens but also provide a valuable foundation for future studies on limb malformations and genetic improvement in poultry. Future functional validation experiments will further confirm the specific biological roles of these candidate genes and may promote the development of novel genetic improvement strategies.

## Data Availability

Our sequencing data have been deposited in the National Center for Biotechnology Information (NCBI) Sequence Read Archive (SRA) under the BioProject accession number PRJNA1147455.
